# Caring for Patients Without Documentation Status: What Motivates Us and Sustains Us

**DOI:** 10.1007/s10903-021-01280-x

**Published:** 2021-09-28

**Authors:** Dahlia A. Kaki, Anjali Dutt, Riham M. Alwan

**Affiliations:** 1 School of Medicine, University of California, San Francisco, San Francisco, CA USA; 2 Department of Psychology, University of Cincinnati, Cincinnati, OH USA; 3 Division of Emergency Medicine, University of California, San Francisco, San Francisco, CA USA

**Keywords:** Provider well-being, Provider motivations, Provider resilience, Undocumented immigrant health

## Abstract

Restrictive policies and limited resources create challenges for care delivery for patients without documentation status (PWDS). This study explores the motivators and sustainers for healthcare providers serving PWDS. Twenty-four direct providers in public and private sectors were interviewed using semi-structured, in-depth interviews. Two members of the research team independently coded interviews using inductive thematic analysis. Four major themes emerged illustrating intrinsic and extrinsic sources that motivated and sustained providers: (1) a sense of calling to serve their community; (2) solidarity is sustaining; (3) organizational culture as a key element for provider engagement; (4) insight into necessary change. Providers who care for PWDS are driven and sustained by internal motivations and a sense of solidarity in working towards better care access for their marginalized patients. Findings illustrate the importance of recruiting and retaining providers with histories of recent migration. Immigration and healthcare policy reform may improve provider workflow.

## Background

Over three percent of the United States population are people without documentation status (PWDS) [1]. Healthcare providers serve an important role in supporting PWDS by addressing patient’s medical and social needs and aiding navigation of complex immigration challenges [2]. Addressing the structural and procedural challenges associated with supporting PWDS, including lack of public funding, legal complications, and patient fear, can lead to provider burnout which has negative implications for healthcare systems and patients [3].

Providers who work with marginalized populations experience increased risk for burnout and high turnover, further straining limited-resource settings [4]. Provider burnout is linked to lower-quality care, medical errors, and decreased patient satisfaction. At the systems-level, this phenomenon results in decreased productivity, increased provider turnover, and higher expenditures (e.g., medical errors) [5]. Burnout is also associated with a decreased “sense of calling,” a well-documented motivation for providers joining the medical profession, especially those working with marginalized communities [6].

Studies exploring providers’ motivations to work with marginalized populations note several factors: personal connection with underserved communities, feelings of responsibility and fulfillment, and qualification for loan-repayment programs [7, 8]. However, work with underserved populations often contends with increased administrative responsibilities, larger patient panels, and complex patient needs, all documented risk factors for burnout [9].

Studies exploring the relationship between provider burnout and caring for underserved populations highlight the frustration of being unable to meet their patients’ social needs, which often are inextricably linked to patients’ health outcomes [10–13]. Political and economic policies that deprive marginalized populations of education, food access, housing, employment, etc. lead to preventable illnesses. Providers serving PWDS often work in safety-net healthcare systems, infrastructures that are historically underfunded, under-resourced, and under-staffed. The result is a larger population of healthcare-seeking patients flooding a healthcare system that is not designed to meets the root cause of their needs.

## Conceptual Framework

Despite challenges, many providers working on the frontlines have developed resilience-building skills and tools to ward off burnout, including strong interpersonal and professional relationships, boundary-setting, and self-care [14]. Nevertheless, no studies explore the specific motivators and sustainers for providers who care for PWDS. The current study is influenced by theories and values from action research, which both underscore that communities impacted by a specific issues are best equipped with insight to address the issue and encourage research that is grounded in identifying actions that create needed change [15]. Developing a better understanding of why providers seek to work with PWDS and the resources and skills that allow them to succeed in these roles is critical for healthcare systems to address lacunae and devote appropriate support to providers and, in turn, their patients. This qualitative study seeks to address this gap and explore what motivates and sustains providers who care for PWDS in San Francisco Bay Area.

## Methods

The University of California, San Francisco IRB approved this study as a non-human subjects’ determination.

### Participants

Participants were healthcare providers residing in San Francisco Bay Area who were recruited via snowball sampling. Twenty-four employed adults with three to forty years of experience working with PWDS were included in this study (Table 1). Fifteen of the participants were women. Additionally, 66% (N = 16) were immigrants from several countries including Afghanistan, Mexico, and Vietnam, with the majority of Latinx descent. Inclusion criteria were: experience providing direct care for PWDS and affiliation with a community-based organization, school, or health center that served PWDS. Participants who did not speak English were excluded. Ethical standards were upheld through the use of informed consent, confidentiality, and ability to withdraw at any point.

**Table.1:** Participant demographics

ID	Medical specialty/profession	Academic title	Gender	Years of practice	Practice zip code	Immigrant?
1	Internal Medicine	Professor	F	18	94110	Y
2	Family and Community Medicine	Assistant Professor	M	5	94124, 94110	Y – first gen
3	Family and Community Medicine	Fellow Physician	F	5	94110	Y
4	Emergency Medicine	Assistant Professor	F	4	94110	N
5	Pediatrics	Clinical Professor	F	10	94609	Y
6	Pediatrics	Assistant Professor	F	13	94110	N
7	Pediatrics	Clinical Professor	F	29	94609	Y
8	Community Health Center Administration	Executive Director	F	26	94110	Y
9	Family and Community Medicine	Professor	F	25	94118	N
10	Internal Medicine	Professor; Associate Dean	M	40	94143	Y – first gen
11	Pediatrics	Associate Professor	M	14	94110	Y – first gen
12	Anesthesia	Professor; Vice Dean	F	39	94110	N
13	Licensed Marriage and Family Therapist (Public School Health Center)	N/A	F	20	94605	Y
14	Social Worker	N/A	F	30	94609	N
15	Social Worker	N/A		14		Y
16	Internal Medicine/Nephrology	Assistant Professor	F	8	94143	Y
17	Pediatrics, Neurology	Assistant Professor	F	6		N
18	Medical Education and Community Health Center Adinistration	Associate Director of Medical Education	F	10	95343, 93701	Y
19	Community Health Center Administration, Mental Health Case Management	N/A	M	6	94110	Y
20	Community Health Center Administration	Chief Deputy of Administration, Programs	F	10	94607	Y – first gen
21	Community Health Center Provider: Pediatrics, Primary Care	Attending Physician	F	8	94601	N
22	Social Worker (Public School)	N/A	F	3	94605	N
23	Internal Medicine	Professor	M	Unknown	95817	Y
24	Education and Mental Health Research	N/A	M	32	95817	Y

### Data Collection

Semi-structured interviews, ranging from 45 to 60 min, were conducted in English by two investigators between May and July 2020. The open-ended interview guide was developed and vetted by field experts and direct providers (Appendix A). Interviews explored the motivators and sustainers for providers working with PWDS. Interviews were conducted in English on a password-protected virtual platform and audio-recorded. Interviews were subsequently transcribed and reviewed for accuracy and content.

### Data Analysis

We used an inductive thematic analysis to analyze the transcripts. Two members of the research team independently reviewed and coded each interview upon completion of data collection, using the support of Dedoose software. Through a series of meetings and transcription analyses, we built a codebook with agreement of the research team. Subsequently, investigators independently placed codes identified in the interviews into broad thematic categories. Through an iterative process and a series of weekly meetings, the group reached consensus on the categories and continued to independently code the interviews. Participant enrollment continued until saturation of themes occurred and no new information came forth, a standard procedure for estimating sample size for qualitative studies [16, 17]. We performed a thorough process of member checking, which involves reviewing the data and the results with participants to check for accuracy and resonance with their experiences, to ensure the credibility and trustworthiness of the findings. Finally, local community stakeholders, national experts, and study participants reviewed the emergent themes of this study.

## Results

**Fig. 1:**
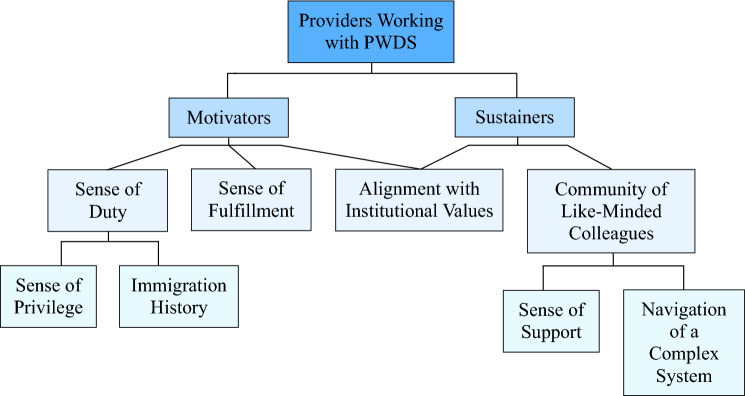
Provider motivators and sustainers concept map

Overwhelmingly, providers expressed pride and satisfaction in their work with PWDS. Providers also expressed significant physical and emotional challenges from working in limited-resource settings, substantial administrative duties, and dynamic healthcare policies (Fig. 1). Our results detail four major themes illustrating intrinsic and extrinsic sources that motivated and sustained their work, despite the challenges: (1) a sense of calling to serve the community; (2) solidarity that created sustenance; (3) organizational culture; and (4) insight into necessary change.

### Theme 1: Calling to Serve the Community

Overwhelmingly, participants described an intrinsic motivation to advocate and provide quality care for immigrants. This “calling to serve” was deeply rooted in a desire to support those who are most in need, yet simultaneously unlikely to have those needs adequately addressed by the healthcare system. For example, one participant explained their interest in working with PWDS:

“To help those who need help. It ends up being those who are on the margins, those need the most help….Everybody here has that same commitment. It’s our core, it’s our religion...the ability to use our MD-ness to advocate.” (Participant 7)

Many participants explained that their motivation derived from their own, or their families’, immigration history. They described a sense of duty linked to feeling privileged as a provider, while simultaneously having intimate knowledge of the barriers and hardship faced by immigrants:

“Immigration has always been something that I think about a lot, just from my own personal narratives and also just contrasting my life with my [Mexican] cousin’s life, my brilliant cousins who just haven't had the opportunity...And that's why I am dedicated to this work. I think about my family a lot, about some of the most brilliant people I've ever met, who just didn't have access to those opportunities.” (Participant 3)

Additionally, shared immigration history gave providers greater empathy with and understanding of the challenges their patients faced. It further motivated providers to do for others what they wished they could do for their own families. Providers were driven by the pride they felt in serving communities to which they were deeply connected. Patients were likened to family members, further establishing providers’ duty to serve them:

“There’s something very rewarding and empowering in working in the same communities that you arose from. And so for me, working in [community-based clinic] and seeing that the patients are very similar to my own family... again and again, we hear from our doctors that, gosh, as hard as the work is, because it's literally like working with your family, which is hard, it also feels like you see the direct impact that you have” (Participant 20)

Overall, a sense of calling strongly explained why participants opted to work with PWDS, despite the myriad of challenges associated with this work. Importantly, the majority of providers interviewed drew direct connections between their work and their lived experience outside of their workplace. For many, this connection was rooted in family or personal history of immigration. For others, it had to do with using their work to contribute to needed change in society at large.

### Theme 2: Solidarity is Sustaining

A second theme identified was the sense of solidarity participants felt with others engaged in this work which contributed to sustaining their commitment. These relationships provided a support system of people who could relate and help when challenges arose and allowed providers to do their work with an increased sense of joy. Importantly, these relationships were rooted in shared goals and experiences, allowing for relatability and interpersonal support in various forms.

Many participants described seeking out a group of like-minded colleagues and explained that this group served as a source of energy and strength. For some, "finding their people," was an intentional decision to create a sense of belonging and support. One participant explained:

“Building your gang is really critical…we've formed such a tight friendship and that's helped a lot in being able to process emotions and times when you're short-fused, to be able to ask for permission to complain and not be at your best in front of other people. Those are really important spaces to build.” (Participant 20)

This participant highlighted how having deep friendships with others who can relate to the work they were doing allowed them to fully express and process the emotions they experienced. Researchers in psychology note the importance of experiencing a sense of community and solidarity when cultivating resilience in challenging contexts [18]. For providers working with PWDS, these relationships serve both as a coping mechanism and a source of shared encouragement:

“My coworkers are amazing. All the folks that work in my clinic. They’re dedicated to our families, and they go above and beyond all the time and we really support each other.” (Participant 5)

Others highlighted how feeling a part of a community rooted in shared goals provided a safe space to navigate the complicated structural and organizational challenges associated with working with PWDS. More specifically, the co-worker community gave providers access to a network of people willing to “jerry-rig” the complicated healthcare system:

“We are also in another bubble of providers and like-minded people that are here. We definitely have people who are fighting for access to housing and care…people who are really like true and very incredible advocates… there's a lot of programs that are very inclusive here for populations that are traditionally marginalized.” (Participant 16)

In summary, all of the participants interviewed emphasized the value of having a supportive community anchored in a shared mission. These relationships enabled the participants to feel supported and connected and aided in sustaining their involvement in this work.

### Theme 3: Organizational Culture

A third theme evident in the interviews was the role of workplace culture in both motivating and sustaining providers’ involvement in work with PWDS. This included both appreciating factors such as the size and structure of the organization, as well as the values that drove decision making in the workplace.

One participant explained how important the organizational culture was in making their workplace one where they found joy in work:

“I feel lucky because I’m in this very magical, wonderful [workplace]. And I don’t know if I could do the same work at a bigger [workplace], where we didn’t have that relationship and the culture. The culture of the organization... that helps a lot.” (Participant 22)

Workplace culture served as a strong motivator that could make the hardships associated with this work worthwhile. Other participants highlighted how the alignment of their values with those of institutional leadership and a workplace culture focused on social justice motivated their efforts to to provide improved care to PWDS, despite challenges.

“When I work here, I feel very aligned with the values of the leadership. I feel that if I make a suggestion, and they say ‘No we can’t do that,’ they’re not saying that because our values don’t align, they’re saying that for some other impediment...People who work in this system... want to work in this system” (Participant 1)

### Theme 4: Insight into Necessary Change

All of the individuals interviewed for this study were clinician-experts in providing healthcare to immigrants and PWDS. In addition to factors that motivated and sustained their involvement in work with this community, providers also explained improvements that could be made in the current system of healthcare in which they operate that would improve the quality of care to PWDS (Table 2). To begin, there was unanimous consensus about the need for increased resources in practice settings serving PWDS, namely increased interpreter services, social services, and care coordination.

**Table.2:** Expert recommendations

Descriptor	Quote
Increasing provider resources	“[Put] resources into making sure that access and that foundation of trust is being supported. So, that's language services, transportation, navigation here on the campus.”
	“Invest in the staffing that’s needed to deal with and is very critical to ensuring health equity.”
	“[Increase] cultural competency that pays attention to the culture as well as the other social determinants of health.”
	“[Invest] in medical-legal partnerships that help support undocumented patients.”
Emphasizing community engagement	“[Invest] in workforces that look like the people they serve.”
	“See and recognize and appreciate the good, the productivity, the happiness, the resilience, the innovation [in the communities we serve.”
	“[Address] the fear and [don’t] police people, be pro-asylum, some of these very basic things that make communities feel safe.”
Changing public policy	“[Abolish] this idea of legality or this idea of class of citizen with respect to health.”
	“Universal healthcare is the answer for healthcare.”

Many respondents also highlighted that work that is traditionally considered “non-billable” is often work that is critical to improving access to care for PWDS. Time spent building trust in the community, going above-and-beyond to secure patient needs, and spearheading advocacy and outreach efforts are often not recognized as “productive time.” Participants also explained that time spent connecting with the community allowed providers to develop a more complete understanding of the community which could improve their capacity to offer care.

Lastly, it is critical to note that all respondents advocated for universal healthcare access and immigration reform, despite it being a significantly more challenging goal. However, a healthcare system that supports providers engaged in this work is one that ensures equitable and affordable healthcare access for all patients.

## Discussion

This study documents findings from interviews with healthcare providers who work with PWDS in San Francisco Bay Area, focusing on what motivates and sustains their involvement and identifying areas that could improve the experiences of providers and patients. Providers described deep intrinsic motivation to work with this population, a sense of solidarity with other providers, and supportive workplace cultures as important motivating and sustaining factors. Participants highlighted how structural changes such as increasing resources, changing policies around billable hours, and advocating for universal healthcare could substantially improve the context of healthcare for PWDS.

The findings contribute to the growing literature that documents challenges within healthcare that arise with increasingly anti-immigrant policies in the United States. Previous research illustrates that immigrants are increasingly fearful of engaging with healthcare systems, which leads to delays in care and disenrollment from healthcare and social service programs [19]. Researchers also documented the deleterious impact these policies have had on immigrant health [20–24]. Within this context, our research highlights both challenges healthcare providers are experiencing and sources of support providers receive in entering and sustaining their work with PWDS. In noting how crucial these providers are in addressing the health needs of a particularly marginalized community, these findings yield important insights for recruiting and supporting valuable employees.

Overwhelmingly, providers exhibited a strong intrinsic motivation to be engaged in this work. Although the call for this study was not limited by immigration status, the majority of respondents had recent immigration histories. It is perhaps not surprising that many of the providers were able to connect their drive for this work to their own backgrounds. The findings highlight the value of recruiting and continuously supporting providers with family histories of recent migration who can relate to the struggles of their patients.

The findings also yield insights that could incentivize changes within organizations. Providers discussed the value of feeling a sense of solidarity with their co-workers and others connected to the work. Healthcare settings should consider hiring cohorts, as opposed to individuals, who share a passion for this work, can become a supportive network for each other, and can help sustain collective passion and drive. Employers should create regular and safe spaces for connection and knowledge sharing among providers who work with PWDS. This can reduce the possibility of feeling isolated when engaging in the challenges of this work, open up space to share personal knowledge and insight into related topics, and allow providers to share frustrations that arise with others who can relate to their work.

The findings also encourage structural changes that would improve healthcare for PWDS and the workplace experiences of their providers. First, changing pay structures that require work to be contained to “billable” hours could allow for more relationship building between provider and patient, creating deeper understandings of the social and health context of the patients and likely lead to the provision of better healthcare. Second, all participants advocated for structural changes at a policy level, namely universal healthcare and immigration reform. Individuals who are committed to creating better healthcare realities for PWDS and their providers should lobby for these changes to address concerns of systemic injustice that lead to burnout.

### Limitations

This study analyzed the perspective and opinions of individuals who remain in this line of work, despite the challenges. It would be equally helpful to hear from providers who have left the field to gain insight into topics such as breaking points that catalyzed their exit and perspectives on factors that would have supported their continuation. It is also important to explore the experiences of providers working in different regions where they may encounter more barriers to this work. Finally, it is important to note that snowball sampling often leads to the recruitment of like-minded participants—further research could explore this topic using different recruitment methodologies.

## New Contributions to the Literature

This study highlights the critical need to invest in and support healthcare providers working with PWDS. The findings reinforce the need for widespread healthcare infrastructure changes, with specific changes allow these providers to excel in their work. This includes recruiting and championing providers with immigrant backgrounds, providing adequate compensation and recognition of non-traditional work activities, and carving spaces for collaboration and wellness amongst such providers.

## References

[1] Budiman A. Key findings about U.S. immigrants. Pew Research Center. https://www.pewresearch.org/fact-tank/2020/08/20/key-findings-about-u-s-immigrants/. Published 2021. Accessed August 16, 2021.

[2] Valentín-Cortés M, Benavides Q, Bryce R (2020). Application of the minority stress theory: understanding the mental health of undocumented latinx immigrants. Am J Community Psychol.

[3] Misra-Hebert AD, Kay R, Stoller JK (2004). A review of physician turnover: rates, causes, and consequences. Am J Med Qual.

[4] Maslach C, Leiter MP (2016). Understanding the burnout experience: recent research and its implications for psychiatry. World Psychiatry.

[5] West CP, Dyrbye LN, Shanafelt TD (2018). Physician burnout: contributors, consequences and solutions. J Intern Med.

[6] Getzin A, Bobot BL, Simpson D (2016). Sustaining family physicians in urban underserved settings. Fam Med.

[7] Jager AJ, Tutty MA, Kao AC (2017). Association between physician burnout and identification with medicine as a calling. Mayo Clin Proc.

[8] Curlin FA (2006). Following the call: how providers make sense of their decisions to work in faith-based and secular urban community health centers. J Health Care Poor Underserved.

[9] Odom Walker K (2010). Recruiting and retaining primary care physicians in urban underserved communities: the importance of having a mission to serve. Am J Public Health.

[10] Hayashi AS, Selia E, McDonnell K (2009). Stress and provider retention in underserved communities. J Health Care Poor Underserved.

[11] Cervantes L (2018). Clinicians’ perspectives on providing emergency-only hemodialysis to undocumented immigrants: a qualitative study. Ann Intern Med.

[12] Munnangi S (2018). Burnout, perceived stress, and job satisfaction among trauma nurses at a Level I Safety-Net Trauma Center. J Trauma Nurs.

[13] Olayiwola JN (2018). Higher perceived clinic capacity to address patients’ social needs associated with lower burnout in primary care providers. J Health Care Poor Underserved.

[14] Zwack J, Schweitzer J (2013). If every fifth physician is affected by burnout, what about the other four? Resilience strategies of experienced physicians. Acad Med.

[15] Dutt A, Jacquez F, Chaudhary N (2021). Creating collective solidarity: Insights from the development and process evaluation of civic action for refugee empowerment in Cincinnati. Cultur Divers Ethnic Minor Psychol.

[16] Davis SN (2018). Recruitment techniques and strategies in a community-based colorectal cancer screening study of men and women of African Ancestry. Nurs Res.

[17] Corbin JM, Strauss AL (2015). Basics of qualitative research: techniques and procedures for developing grounded theory.

[18] Dutt A (2018). Feminist organizing in rural Nicaragua: assessing a psychosocial process to promote empowered solidarity. Am J Community Psychol.

[19] Page KR, Polk S (2017). Chilling effect? Post-election health care use by undocumented and mixed-status families. N Engl J Med.

[20] Fleming PJ (2019). A qualitative study on the impact of the 2016 US election on the health of immigrant families in Southeast Michigan. BMC Public Health.

[21] Lopez W (2019). Separated: family and community in the aftermath of an immigration raid.

[22] Castañeda H (2019). Borders of belonging struggle and solidarity in mixed-status immigrant families.

[23] Kline N (2018). Pathogenic policing immigration enforcement and health in the U.S. South.

[24] Benavides Q, Doshi M, Valentín-Cortés M (2021). Immigration law enforcement, social support, and health for Latino immigrant families in Southeastern Michigan. Soc Sci Med.

